# Supplementary figures and images for: Clinical value of Lipoprotein(a) combined with CatLet coronary score in predicting adverse events after emergency PCI for AMI patients
Source: PLoS One. 2026 Feb 23;21(2):e0342704. doi: 10.1371/journal.pone.0342704 (PMC12928423; doi:10.1371/journal.pone.0342704)

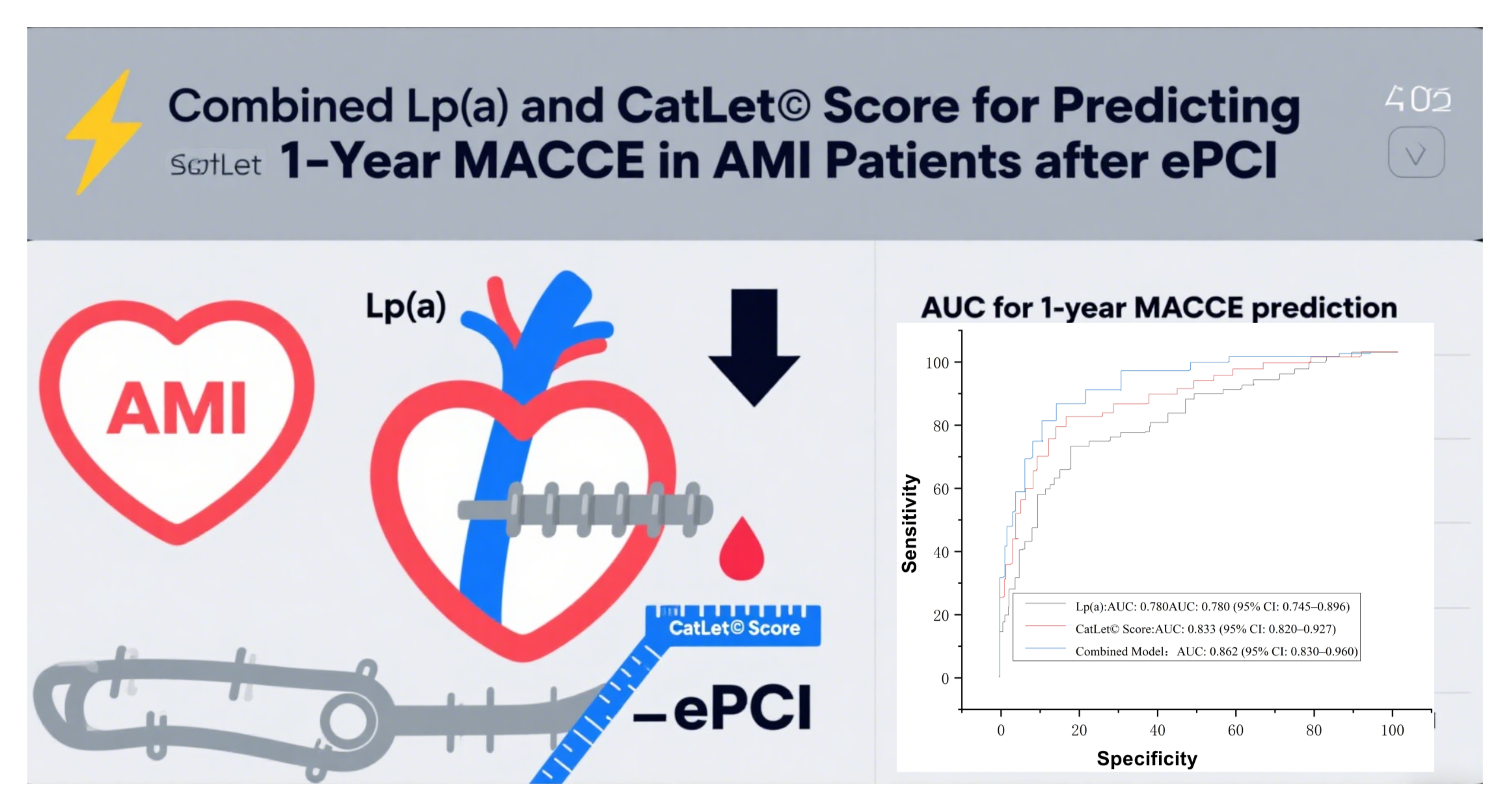

Supplement: S1 File — (TIFF) [file pone.0342704.s001.tiff]
