# Supplementary material for: Clinical value of Lipoprotein(a) combined with CatLet coronary score in predicting adverse events after emergency PCI for AMI patients
Source: PLoS One. 2026 Feb 23;21(2):e0342704. doi: 10.1371/journal.pone.0342704 (PMC12928423; doi:10.1371/journal.pone.0342704)
Supplement: Attachment — Submitted filename: PONE-D-25-57401-RB.pdf [file pone.0342704.s002.pdf]

PLOS ONE

Clinical Value of Lipoprotein(a) Combined with CatLet© Coronary Score in Predicting Adverse Events after Emergency PCI for AMI Patients

--Manuscript Draft--

**Manuscript Number:**	PONE-D-25-57401
**Article Type:**	Research Article
**Full Title:**	Clinical Value of Lipoprotein(a) Combined with CatLet© Coronary Score in Predicting Adverse Events after Emergency PCI for AMI Patients
**Short Title:**	Lp(a) and CatLet© Score in Predicting Adverse Events after AMI-PCI
**Corresponding Author:**	sheng tu<br>Bozhou Hospital Affiliated to Anhui Medical University: The People's Hospital of Bozhou<br>Bozhou, CHINA
**Keywords:**	Lipoprotein(a); CatLet© coronary score; acute myocardial infarction; percutaneous coronary intervention; Major adverse cardiovascular and cerebrovascular events; Prognostic assessment
**Abstract:**	Background and aims<br><br>Lipoprotein(a) [Lp(a)] promotes atherosclerotic plaque vulnerability through pro-inflammatory and thrombogenic pathways, while the CatLet© angiographic score quantifies coronary lesion complexity. We hypothesized that their integration would improve prognostication in acute myocardial infarction (AMI) after emergency percutaneous coronary intervention (ePCI).<br><br>Methods<br><br>In this retrospective cohort, 307 AMI patients undergoing successful ePCI (2020–2022) were stratified by 1-year major adverse cardiovascular/cerebrovascular events (MACCE). Serum Lp(a) and troponin I were measured post-admission. CatLet© and Gensini scores were assessed by blinded analysts. Multivariable logistic regression and ROC analyses evaluated predictive performance.<br><br>Results<br><br>MACCE patients (n=78) exhibited higher Lp(a) (135.99±33.07vs. 123.35±42.70nmol/L, P=0.0178) and CatLet© scores (33.58±9.04vs. 30.80±8.24, P=0.0012) versus controls. Lp(a) (OR=2.339,95%CI:1.519–3.603, P<0.001) and CatLet© score (OR=1.092, 95%CI:1.027–1.161, P=0.005) independently predicted MACCE. The combined model Lp(a)≥70.70 nmol/L + CatLet©≥18.6) significantly outperformed individual markers (AUC 0.862 [95%CI:0.83–0.96] vs. 0.780/0.833; DeLong's test confirmed the superiority of the combined model over individual predictors (P = 0.0089, Z = 2.64 vs. Lp(a); P = 0.034, Z = 2.12 vs. CatLet© score), with 88% sensitivity and 83% specificity.<br><br>Conclusions<br><br>The Lp(a)-CatLet© synergy enhances MACCE risk stratification in ePCI-treated AMI, reflecting complementary pathobiological (Lp(a)-driven plaque vulnerability) and anatomical (CatLet©-quantified complexity) pathways. This dual-parameter approach could support post-PCI risk stratification and follow-up planning.
**Order of Authors:**	Mengru Wang
	sheng tu
	Fudong Hu
	Rongyan Jiang
**Additional Information:**	

Powered by Editorial Manager® and ProduXion Manager® from Aries Systems Corporation

Question	Response
**Financial Disclosure**<br><br>Enter a financial disclosure statement that describes the sources of funding for the work included in this submission. Review the submission guidelines for detailed requirements. View published research articles from *PLOS ONE* for specific examples.<br><br>This statement is required for submission and **will appear in the published article** if the submission is accepted. Please make sure it is accurate.<br><br>**Funded studies**<br>Enter a statement with the following details:<br>• Initials of the authors who received each award<br>• Grant numbers awarded to each author<br>• The full name of each funder<br>• URL of each funder website<br>• Did the sponsors or funders play any role in the study design, data collection and analysis, decision to publish, or preparation of the manuscript?<br><br>Did you receive funding for this work?	The author(s) received no specific funding for this work.
**Competing Interests**<br><br>Use the instructions below to enter a competing interest statement for this submission. On behalf of all authors, disclose any competing interests that could be perceived to bias this work—acknowledging all financial support and any other relevant financial or non-financial competing interests.<br><br>This statement is required for submission and **will appear in the published article** if the submission is accepted. Please make sure it is accurate and that any funding sources listed in your Funding Information later in the submission form are also	The authors have declared that no competing interests exist.

declared in your Financial Disclosure statement.<br><br>View published research articles from *PLOS ONE* for specific examples.<br><br>**NO authors have competing interests**<br><br>Enter: *The authors have declared that no competing interests exist.*<br><br>**Authors with competing interests**<br><br>Enter competing interest details beginning with this statement:<br><br>*I have read the journal's policy and the authors of this manuscript have the following competing interests: [insert competing interests here]*<br><br>* typeset	
**Ethics Statement**<br><br>Enter an ethics statement for this submission. This statement is required if the study involved:<br><br>• Human participants<br>• Human specimens or tissue<br>• Vertebrate animals or cephalopods<br>• Vertebrate embryos or tissues<br>• Field research<br><br>Write "N/A" if the submission does not require an ethics statement.<br><br>General guidance is provided below. Consult the submission guidelines for detailed instructions. **Make sure that all information entered here is included in the Methods section of the manuscript.**	N/A

Powered by Editorial Manager® and ProduXion Manager® from Aries Systems Corporation

**Format for specific study types**<br><br>**Human Subject Research (involving human participants and/or tissue)**<br>• Give the name of the institutional review board or ethics committee that approved the study<br>• Include the approval number and/or a statement indicating approval of this research<br>• Indicate the form of consent obtained (written/oral) or the reason that consent was not obtained (e.g. the data were analyzed anonymously)<br><br>**Animal Research (involving vertebrate animals, embryos or tissues)**<br>• Provide the name of the Institutional Animal Care and Use Committee (IACUC) or other relevant ethics board that reviewed the study protocol, and indicate whether they approved this research or granted a formal waiver of ethical approval<br>• Include an approval number if one was obtained<br>• If the study involved *non-human primates*, add *additional details* about animal welfare and steps taken to ameliorate suffering<br>• If anesthesia, euthanasia, or any kind of animal sacrifice is part of the study, include briefly which substances and/or methods were applied<br><br>**Field Research**<br><br>Include the following details if this study involves the collection of plant, animal, or other materials from a natural setting:<br>• Field permit number<br>• Name of the institution or relevant body that granted permission	
**Data Availability**<br><br>Authors are required to make all data underlying the findings described fully available, without restriction, and from the time of publication. PLOS allows rare exceptions to address legal and ethical concerns. See the PLOS Data Policy and FAQ for detailed information.	Yes - all data are fully available without restriction

A Data Availability Statement describing where the data can be found is required at submission. Your answers to this question constitute the Data Availability Statement and **will be published in the article**, if accepted.<br><br>**Important:** Stating 'data available on request from the author' is not sufficient. If your data are only available upon request, select 'No' for the first question and explain your exceptional situation in the text box.<br><br>Do the authors confirm that all data underlying the findings described in their manuscript are fully available without restriction?	
**Describe where the data may be found in full sentences. If you are copying our sample text, replace any instances of XXX with the appropriate details.**<br><br>• If the data are **held or will be held in a public repository**, include URLs, accession numbers or DOIs. If this information will only be available after acceptance, indicate this by ticking the box below. For example: *All XXX files are available from the XXX database (accession number(s) XXX, XXX.*).<br>• If the data are all contained **within the manuscript and/or Supporting Information files**, enter the following: *All relevant data are within the manuscript and its Supporting Information files.*<br>• If neither of these applies but you are able to provide **details of access elsewhere**, with or without limitations, please do so. For example:<br><br>*Data cannot be shared publicly because of [XXX]. Data are available from the XXX Institutional Data Access / Ethics Committee (contact via XXX) for researchers who meet the criteria for access to confidential data.*<br><br>*The data underlying the results presented in the study are available from (include the name of the third party*	All relevant data are within the manuscript and its Supporting Information files.

and contact information or URL).<br>• This text is appropriate if the data are owned by a third party and authors do not have permission to share the data.<br><br>* typeset	
Additional data availability information:	

Powered by Editorial Manager® and ProduXion Manager® from Aries Systems Corporation

manuscript Click here to access/download;Manuscript;manuscript - 2025-10-23.docx

Title Page

Title: Clinical Value of Lipoprotein(a) Combined with CatLet© Coronary Score in Predicting Adverse Events after Emergency PCI for AMI Patients

Running title: Lp(a) and CatLet© Score in Predicting Adverse Events after AMI-PCI

Authors: Mengru Wang[1], Fudong Hu[2],Rongyan Jiang[1],Sheng Tu[1,*] MD, PhD;

Affiliation:

1. Mengru Wang, Department of Cardiology, Bozhou Hospital Affiliated to Anhui Medical University, Bozhou; No. 616 Duzhong Road, Qiaocheng District, Bozhou City, Anhui Province, China.

2. Fudong Hu, Department of Cardiology, Department of Cardiology, The First Affiliated Hospital of Zhengzhou University, Zhengzhou, Henan, PR China.

3. Rongyan Jiang, Department of Cardiology, Bozhou Hospital Affiliated to Anhui Medical University, Bozhou People's Hospital; No. 616 Duzhong Road, Qiaocheng District, Bozhou City, Anhui Province, China.

4. Sheng Tu, Department of Cardiology, Bozhou Hospital Affiliated to Anhui Medical University,No. 616 Duzhong Road, Qiaocheng District, Bozhou City, Anhui Province, China.

5. ∗Corresponding Author

Correspondence to Professor Dr. Sheng Tu, MD, PhD

Address: No. 616 Duzhong Road, Qiaocheng District, Bozhou City, Anhui Province, China

Postal Code: 236800

6. Email: tusheng77@163.com

Cell phone: +86 18756796120

Telephone: +86 558 5675301

Fax: +86 558 5675301

Clinical Value of Lipoprotein(a) Combined with CatLet© Coronary Score in Predicting Adverse Events after Emergency PCI for AMI Patients

Abstract

Background and aims: Lipoprotein(a) [Lp(a)] promotes atherosclerotic plaque vulnerability through pro-inflammatory and thrombogenic pathways, while the CatLet© angiographic score quantifies coronary lesion complexity. We hypothesized that their integration would improve prognostication in acute myocardial infarction (AMI) after emergency percutaneous coronary intervention (ePCI).

Methods: In this retrospective cohort, 307 AMI patients undergoing successful ePCI (2020－2022) were stratified by 1-year major adverse cardiovascular/cerebrovascular events (MACCE). Serum Lp(a) and troponin I were measured post-admission. CatLet© and Gensini scores were assessed by blinded analysts. Multivariable logistic regression and ROC analyses evaluated predictive performance.

Results: MACCE patients (n=78) exhibited higher Lp(a) (135.99±33.07vs.

123.35±42.70nmol/L, P=0.0178) and CatLet© scores (33.58±9.04vs. 30.80±8.24, P=0.0012) versus controls. Lp(a) (OR=2.339,95%CI:1.519−3.603, P < 0.001) and CatLet© score (OR=1.092, 95%CI:1.027–1.161, P=0.005) independently predicted MACCE. The combined model Lp(a)⩾70.70 nmol/L + CatLet©⩾18.6) significantly outperformed individual markers (AUC 0.862 [95%CI:0.83−0.96] vs. 0.780/0.833; DeLong's test confirmed the superiority of the combined model over individual predictors (P = 0.0089, Z = 2.64 vs. Lp(a); P = 0.034, Z = 2.12 vs. CatLet© score), with 88% sensitivity and 83% specificity.

Conclusions: The Lp(a)-CatLet© synergy enhances MACCE risk stratification in ePCI-treated AMI, reflecting complementary *pathobiological* (Lp(a)-driven plaque vulnerability) and *anatomical* (CatLet©-quantified complexity) pathways. This dual-parameter approach could support post-PCI risk stratification and follow-up planning.

Keywords:

Lipoprotein(a); CatLet© coronary score; Acute myocardial infarction; Percutaneous coronary intervention; Major adverse cardiovascular and cerebrovascular events; Prognostic assessment

1.Introduction

Accurate risk stratification following emergency revascularization for AMI remains a critical challenge in contemporary cardiology practice. While current prognostic tools incorporating biochemical markers (e.g., cardiac troponins) and angiographic indices (e.g., SYNTAX score) provide modest predictive value(1, 2), their individual

limitations—including biological variability and anatomical oversimplification—underscore the need for more robust multimodal approaches(3, 4).

Emerging evidence implicates lipoprotein(a) (Lp(a)), a proatherogenic and prothrombotic particle, as a key mediator of post-PCI complications(5). Concurrently, the CatLet© scoring system (www.catletscore.com) has demonstrated superior discriminatory capacity for coronary lesion complexity compared to conventional scoring systems by incorporating dynamic weighting for bifurcation lesions, calcification, and functional significance(6).

We hypothesized that integrating Lp(a)'s pathobiological insights with CatLet©'s anatomical precision would optimize MACCE prediction. This investigation represents the first evaluation of this dual-parameter model in ePCI-AMI patients.

2. Materials and Methods

2.1 Study Design and Population

This retrospective cohort study included 307 consecutive acute myocardial infarction (AMI) patients undergoing successful emergency percutaneous coronary intervention (ePCI) at Bozhou People's Hospital (China) between January 2020 and May 2022. AMI was diagnosed per the Fourth Universal Definition of Myocardial Infarction. Key exclusion criteria: prior MI, chronic heart failure (LVEF <40%), severe renal dysfunction (eGFR <30 mL/min/1.73m^2), or non-atherosclerotic coronary disease (e.g., vasculitis, cardiomyopathy). The study was conducted in accordance with the

Declaration of Helsinki. The protocol approval was obtained from the Medical Ethics Committee of Bozhou Hospital affiliated with Anhui Medical University(No. bz-2020-004). The identity of patients remained anonymous, and the requirement for informed consent was waived due to the retrospective anonymized data analysis.

2.2 Procedural and Pharmacological Protocols

All patients received guideline-directed therapy:

Pre-PCI: Loading doses of aspirin (300 mg) plus P2Y12 inhibitor (clopidogrel 300/600 mg or ticagrelor 180 mg).

Anticoagulation: Intravenous unfractionated heparin (100 IU/kg; ACT >300 sec), with glycoprotein IIb/IIIa inhibitors at operator discretion.

Post-PCI: Dual antiplatelet therapy (aspirin 100 mg/day + clopidogrel 75 mg/day or ticagrelor 90 mg twice daily) for ⩾12 months, with high-intensity statins and beta-blockers as indicated.

High-risk support: Intra-aortic balloon pump (IABP) or IVUS-guided PCI for complex lesions (e.g., severe calcification, bifurcation).

2.3 Biomarker and Angiographic Analysis

Lp(a) quantification: Fasting serum Lp(a) was measured 24h post-admission via immunoturbidimetric assay (Roche; interassay CV <5%) on a Roche (cobas c 501 analyzer).(On the morning of the second day after admission, venous blood was drawn from the patient(7). Blood sample preparation involved drawing approximately 4 mL

of venous blood from the median cubital vein under sterile conditions, after the patient had fasted overnight. The sample was allowed to stand for 20 minutes before being centrifuged at 4000 rpm to separate the serum. The serum was then stored at -80°C for subsequent measurement of Lp(a) concentration.)

2.4 Angiographic scoring:

Gensini Score: Lesion severity was graded based on coronary stenosis degree, with segment-specific weighting factors applied. The total score was derived from the sum of individual lesion scores(8).

CatLet© Coronary Score: Angiographic complexity was assessed using the web-based CatLet© scoring calculator (available at http://www.catletscore.com). Only stenotic lesions were quantified(6, 9).

Follow-up

Follow-up was conducted via clinical visits and telephone interviews at 1, 6, and 12 months. Loss to follow-up was <5%. The censoring date was May 31, 2023**,** with yearly assessments continuing thereafter. A routine angiographic evaluation was advised at the 12-month mark following PCI. If patients experienced a return of angina symptoms, angiography was conducted earlier. For those who did not follow the recommended follow-up schedule, telephone interviews were conducted(10).

2.5 Endpoint Definitions(10, 11)

The main outcome of this research was a patient-focused composite measure of major adverse cardiac and cerebrovascular events (MACCE) at 1-year, encompassing all-cause mortality, myocardial infarction (MI), and revascularization of the target vessel

or lesion (TVR/TLR). When evaluating cumulative outcomes, each event was counted only once, prioritizing the first occurrence.

The secondary outcomes included a safety composite endpoint of all-cause death, MI, and stroke, alongside individual MACCE components and stent thrombosis (ST).

Death was categorized as any post-procedure fatality, classified into cardiac or non-cardiac based on the Academic Research Consortium (ARC) guidelines. A death was presumed to be of cardiac origin unless proven otherwise. Cardiac death encompassed fatalities due to cardiac issues (e.g., MI, low-output heart failure, lethal arrhythmias), those related to the procedure, or those of unknown origin.

Myocardial infarction (MI) was defined in accordance with the fourth universal MI definition(12), emphasizing elevated cardiac troponin (cTn) levels exceeding the 99th percentile upper reference limit (URL), symptoms of myocardial ischemia, ECG changes, and angiographic findings. Within 48 hours post-procedure, cTn levels exceeding 5 times the 99th percentile URL after PCI or 10 times after CABG indicated periprocedural MI in patients with normal baseline cTn levels. If baseline levels were elevated, stable, or decreasing, a cTn increase of over 20% also signified periprocedural PCI-related MI. Q-wave MI was identified by the presence of a new pathological Q-wave in at least two contiguous leads after initial treatment.

TVR was any surgical or percutaneous revascularization of any segment within the stented vessel (including target lesions and adjacent branches) within a year, such as the left main, left anterior descending, and left circumflex arteries. A pre-planned staged PCI was not deemed a TVR.

Definite stent thrombosis (ST) was defined per ARC criteria for PCI, while graft occlusion followed an ARC-like definition for CABG.

The definition of stroke is based on the currently internationally recognized criteria primarily derived from the expert consensus statement of the American Heart Association/American Stroke Association (AHA/ASA)(13).

Core Definition:

Stroke is defined as central nervous system (CNS) cell death accompanied by permanent injury, caused by ischemia or hemorrhage, based on neuropathological, neuroimaging, and/or clinical evidence.

Scope of Coverage:

This includes ischemic stroke (symptomatic CNS infarction), asymptomatic cerebral infarction (CNS infarction without known clinical symptoms), intracerebral hemorrhage, and subarachnoid hemorrhage.

2.6 Statistical Analysis

Continuous variables (mean±SD or median[IQR]) were compared via Student's *t*-test or Mann-Whitney U test; categorical variables (n[%]) via χ^2 or Fisher's exact test. Multivariable logistic regression (adjusted for age, sex, hypertension, diabetes, and LVEF) identified MACCE predictors. ROC analysis determined optimal Lp(a)/CatLet© cutoffs. Statistical significance was set at two-tailed $P<0.05$. Analyses used SPSS v26.0 (IBM).

3.Results

3.1 Baseline Characteristics

The MACCE group exhibited lower LVEF (51.6±11.7% vs. 54.9±12.0%, P=0.038), higher peak cTnI (4.86±2.13 vs. 4.35±1.56 ng/mL, P=0.023), and elevated Lp(a) (135.99±33.07 vs. 123.35±42.70 nmol/L, P=0.018) (Table 1).

3.2 Angiographic characteristics were comparable except for higher CatLet© (33.6±9.0 vs. 30.8±8.2, P=0.001) and Gensini scores (83.9±16.9 vs. 78.5±20.7, P=0.042) in the MACCE group (Table 2).

3.3 Multivariable logistic regression confirmed Lp(a) (OR = 2.339, P < 0.001) and CatLet© score (OR = 1.092, P = 0.005) as independent predictors of MACCE, along with LVEF, cTnI, and Gensini score (Table 3). Consistent results were observed in Cox proportional-hazards analysis (Table 4). Log-transformed Lp(a) as a continuous variable also showed a significant dose-response relationship with MACCE risk (Table 5).

3.4 Predictive Performance of Lp(a) and CatLet© Score for MACCE:in this cohort of AMI patients undergoing emergency PCI, both Lp(a) and the CatLet© coronary angiographic score demonstrated significant prognostic utility for MACCE: Individual Predictors: Lp(a):AUC: 0.780 (95% CI: 0.745–0.896),Optimal cutoff: 70.70 nmol/L (sensitivity 81.0%, specificity 76.5%, P<0.001),Clinical implication: Exceeds the EAS consensus high-risk threshold (125 nmol/L)(7), suggesting potential utility in risk stratification. CatLet© Score:AUC: 0.833 (95% CI: 0.820–0.927),Optimal cutoff: 18.6 points (sensitivity 83.0%, specificity 79.0%,

P<0.001),Anatomic correlation: Aligns with prior validation studies defining scores ⩾18 as indicative of complex coronary anatomy. Combined Model Superiority: The integration of Lp(a) and CatLet© score yielded enhanced predictive performance: AUC: 0.862 (95% CI: 0.830 – 0.960),Sensitivity/specificity: 88.0%/83.0% (P=0.009 vs. individual predictors, DeLong's test confirmed the superiority of the combined model over individual predictors (P = 0.0089, Z = 2.64 vs. Lp(a); P = 0.034, Z = 2.12 vs. CatLet© score).Net reclassification improvement (NRI): 0.35 (95% CI: 0.12 – 0.58; P=0.002),Statistical robustness: Bootstrap validation (1,000 iterations) confirmed model stability (mean AUC ± SD: 0.852 ± 0.021) (Table 6) (Figure 2). The combined model showed excellent calibration across risk deciles (Hosmer-Lemeshow P = 0.422; Table 7).

4. Discussion

This study demonstrates that the integration of lipoprotein(a) [Lp(a)] and the CatLet© coronary angiographic score significantly enhances the prediction of 1-year MACCE in AMI patients undergoing emergency PCI. The combined model outperformed either parameter alone, achieving an AUC of 0.862, with improved sensitivity and specificity. These findings underscore the complementary value of incorporating both a pathobiologic marker of plaque vulnerability and a comprehensive anatomic-functional scoring system into post-PCI risk stratification.

4.1. Synergistic Predictive Value of Lp(a) and CatLet© Score

The independent association of elevated Lp(a) (≥70.70 nmol/L) with MACCE aligns with its well-established proatherogenic, prothrombotic, and proinflammatory

properties (5, 14). Lp(a) promotes plaque instability through mechanisms including the inhibition of fibrinolysis, enhancement of foam cell formation, and induction of endothelial dysfunction. Concurrently, the CatLet© score provided a granular assessment of coronary lesion complexity, incorporating critical features such as bifurcation anatomy, calcification severity, and functional significance—elements not fully captured by traditional scores like SYNTAX or Gensini (6, 9, 15).

The superior predictive performance of the combined model suggests a synergistic interaction between systemic risk (reflected by Lp(a)) and localized anatomic burden (quantified by CatLet©). Patients with high Lp(a) may exhibit a heightened inflammatory and thrombotic milieu, which, when coupled with complex coronary anatomy, predisposes them to recurrent ischemic events. This dual-parameter approach thus bridges a critical gap between circulating risk biomarkers and coronary morphology.

4.2. Comparative Advantages of the CatLet© Score

Our results reinforce the clinical utility of the CatLet© score over conventional angiographic scoring systems. While the Gensini score focuses primarily on stenosis severity, and the SYNTAX score emphasizes lesion complexity, the CatLet© system incorporates dynamic weighting for calcification, angulation, and functional parameters such as TIMI flow—factors directly relevant to PCI difficulty and prognosis (6, 16). As shown in Table 8, the CatLet© score offers a more holistic evaluation, which may explain its stronger discriminatory capacity in our cohort.

4.3. Clinical and Mechanistic Implications

From a pathophysiological perspective, Lp(a) has been implicated in vascular calcification and fibrous cap thinning(17-19), processes that may exacerbate the anatomic complexity quantified by the CatLet© score. This interplay suggests that patients with elevated Lp(a) and high CatLet© scores may represent a high-risk phenotype warranting intensified management. This could include more aggressive lipid-lowering therapy (e.g., PCSK9 inhibitors), prolonged dual antiplatelet therapy, or closer follow-up—though such strategies require validation in prospective trials.

Moreover, the optimal Lp(a) cutoff of 70.70 nmol/L in our cohort is notably lower than the European Atherosclerosis Society consensus threshold of 125 nmol/L (7). This may reflect differences in study populations or the acute clinical context, highlighting the need for context-specific thresholds in AMI settings.

4.4. Limitations

Several limitations should be acknowledged. First, the single-center, retrospective design introduces potential selection bias. Second, although the CatLet© score has been validated in prior studies(6), its application requires specialized training and is not yet widely adopted. Third, Lp(a) assays are not fully standardized across platforms, which may affect the generalizability of the proposed cutoff(19). Finally, unmeasured confounders such as genetic predispositions or residual inflammatory risk may influence outcomes.

4.5. Future Directions

Prospective multicenter studies are needed to validate our findings and refine risk thresholds. Incorporating emerging biomarkers (e.g., high-sensitivity C-reactive

protein) or artificial intelligence–based plaque characterization could further enhance predictive models(20). Additionally, randomized trials are warranted to assess whether risk-guided therapeutic interventions improve outcomes in this high-risk subgroup.

4.6. Conclusion

In conclusion, the combination of Lp(a) and the CatLet© score provides a robust, clinically feasible tool for stratifying MACCE risk in AMI patients after emergency PCI. This integrated model captures both systemic lipid-related risk and coronary anatomic complexity, offering a more comprehensive prognostic assessment than either measure alone. With further validation, this approach could inform personalized secondary prevention strategies and follow-up protocols.

Funding

This study was supported by the National Natural Science Foundation of China (Grant No. 82170333), Bozhou City Key R&D Program of Anhui Province of China (No. bzzc2024004 & bzzc2024001), Medical Science and Technology Research Project of Henan Province (Grant No. SBGJ202102153), Henan Provincial Science and Technology Research Projects (Grant No. 212102310799 and 222102310577).

Conflict of Interest statement

All authors have no conflicts of interest.

Reference

1. Ananthan K, Lyon AR. The Role of Biomarkers in Cardio-Oncology. J Cardiovasc Transl Res. 2020;13(3):431-50.

2. Birnbaum Y, Herz I, Sclarovsky S, Zlotikamien B, Chetrit A, Olmer L, et al. Prognostic

significance of precordial ST segment depression on admission electrocardiogram in patients with inferior wall myocardial infarction. J Am Coll Cardiol. 1996;28(2):313-8.

3. Gholipour A, Shakerian F, Zahedmehr A, Irani S, Malakootian M, Mowla SJ. Bioinformatics Analysis to Find Novel Biomarkers for Coronary Heart Disease. Iran J Public Health. 2022;51(5):1152-60.

4. V R, Rao S, Nayak K, M M, Samanth J, R P. Assessing angiographic results of saline autotransfusion and thrombus aspiration in STEMI patients. Future Cardiol. 2025;21(4):237-43.

5. Minana G, Cordero A, Facila L, Company M, Fernandez-Cisnal A, Valero E, et al. Lipoprotein(a) and Long-Term Recurrent Infarction After an Acute Myocardial Infarction. Am J Cardiol. 2024;211:9-16.

6. He YM, Masuda S, Gao C. Revisit of the CatLet (Hexu) angiographic scoring system: a narrative review. J Thorac Dis. 2023;15(5):2848-58.

7. Mach F, Baigent C, Catapano AL, Koskinas KC, Casula M, Badimon L, et al. 2019 ESC/EAS Guidelines for the management of dyslipidaemias: lipid modification to reduce cardiovascular risk. European heart journal. 2020;41(1):111-88.

8. Rampidis GP, Benetos G, Benz DC, Giannopoulos AA, Buechel RR. A guide for Gensini Score calculation. Atherosclerosis. 2019;287:181-3.

9. Xu M, Wang S, Zhang Y, Zhang J, Ma J, Shen J, et al. Residual coronary artery tree description and lesion EvaluaTion (CatLet) score, clinical variables, and their associations with outcome predictions in patients with acute myocardial infarction. Chin Med J (Engl). 2023;136(20):2459-67.

10. Tu S, Zhang L, Tian Q, Hu F, Wang Y, Chen L. Five-year outcomes of double kissing mini-culotte stenting vs. mini-culotte stenting using drug-eluting stents for the treatment of true coronary bifurcation lesions. Frontiers in cardiovascular medicine. 2024;11:1336750.

11. Lee SH, Hong D, Shin D, Kim HK, Park KH, Choo EH, et al. QFR Assessment and Prognosis After Nonculprit PCI in Patients With Acute Myocardial Infarction. JACC Cardiovascular interventions. 2023;16(19):2365-79.

12. Thygesen K, Alpert JS, Jaffe AS, Chaitman BR, Bax JJ, Morrow DA, et al. Fourth Universal Definition of Myocardial Infarction (2018). Circulation. 2018;138(20):e618-e51.

13. Powers WJ, Rabinstein AA, Ackerson T, Adeoye OM, Bambakidis NC, Becker K, et al. Guidelines for the Early Management of Patients With Acute Ischemic Stroke: 2019 Update to the 2018 Guidelines for the Early Management of Acute Ischemic Stroke: A Guideline for Healthcare Professionals From the American Heart Association/American Stroke Association. Stroke. 2019;50(12):e344-e418.

14. Ting Q, Tian-Yi M, Kang H, Shi-Juan L, Jiang-Hua Z, Jian-Jun LJCAR. Lipoprotein (a)-Related Inflammatory Imbalance: A Novel Horizon for the Development of Atherosclerosis. 2024;26(8).

15. Ming-Xing X, Ruo-Ling T, Terrence D R, Paul S, Thomas B, Roberto DB, et al. The CatLet score: a new coronary angiographic scoring tool accommodating the variable coronary anatomy for the first time. 2020;11(12).

16. Lan-Lin W, Jian-Ping X, Yang H, Heng W, Guo-Zhong Z, Kai W, et al. Coronary Artery Tree Description and Lesion Evaluation (CatLet) score for functional evaluation of

coronary stenosis: a comparison study with pressure wire fractional flow reserve. 2024;14(4).
17. Laura A-H, Julián A-J, Jacob Fog BJFCM. Mechanisms of fibrous cap formation in atherosclerosis. 2023;10(0).
18. Marlys L K, Michael B BJA. Oxidized phospholipid modification of lipoprotein(a): Epidemiology, biochemistry and pathophysiology. 2022;349(0).
19. Jia P, Ming-Ming L, Hui-Hui L, Rui-Xia X, Cheng-Gang Z, Yuan-Lin G, et al. Lipoprotein (a)-mediated vascular calcification: population-based and in vitro studies. 2021;127(0).
20. Jacek KJJCI. Artificial Intelligence-Based Quantitative Coronary Plaque Analysis. 2023;17(3).

Manuscript

Click here to access/download;Manuscript;manuscript - 2025-10-23.pdf

Title Page

Title: Clinical Value of Lipoprotein(a) Combined with CatLet© Coronary Score in Predicting Adverse Events after Emergency PCI for AMI Patients

Running title: Lp(a) and CatLet© Score in Predicting Adverse Events after AMI-PCI

Authors: Mengru Wang[1], Fudong Hu[2],Rongyan Jiang[1],Sheng Tu[1,*] MD, PhD;

Affiliation:

1. Mengru Wang, Department of Cardiology, Bozhou Hospital Affiliated to Anhui Medical University, Bozhou; No. 616 Duzhong Road, Qiaocheng District, Bozhou City, Anhui Province, China.

2. Fudong Hu, Department of Cardiology, Department of Cardiology, The First Affiliated Hospital of Zhengzhou University, Zhengzhou, Henan, PR China.

3. Rongyan Jiang, Department of Cardiology, Bozhou Hospital Affiliated to Anhui Medical University, Bozhou People's Hospital; No. 616 Duzhong Road, Qiaocheng District, Bozhou City, Anhui Province, China.

4. Sheng Tu, Department of Cardiology, Bozhou Hospital Affiliated to Anhui Medical University,No. 616 Duzhong Road, Qiaocheng District, Bozhou City, Anhui Province, China.

5. ∗Corresponding Author

Correspondence to Professor Dr. Sheng Tu, MD, PhD

Address: No. 616 Duzhong Road, Qiaocheng District, Bozhou City, Anhui Province, China

Postal Code: 236800

6. Email: tusheng77@163.com

Cell phone: +86 18756796120

Telephone: +86 558 5675301

Fax: +86 558 5675301

Clinical Value of Lipoprotein(a) Combined with CatLet© Coronary Score in Predicting Adverse Events after Emergency PCI for AMI Patients

Abstract

Background and aims: Lipoprotein(a) [Lp(a)] promotes atherosclerotic plaque vulnerability through pro-inflammatory and thrombogenic pathways, while the CatLet© angiographic score quantifies coronary lesion complexity. We hypothesized that their integration would improve prognostication in acute myocardial infarction (AMI) after emergency percutaneous coronary intervention (ePCI).

Methods: In this retrospective cohort, 307 AMI patients undergoing successful ePCI (2020 – 2022) were stratified by 1-year major adverse cardiovascular/cerebrovascular events (MACCE). Serum Lp(a) and troponin I were measured post-admission. CatLet© and Gensini scores were assessed by blinded analysts. Multivariable logistic regression and ROC analyses evaluated predictive performance.

Results: MACCE patients (n=78) exhibited higher Lp(a) (135.99±33.07vs.

123.35±42.70nmol/L, P=0.0178) and CatLet© scores (33.58±9.04vs. 30.80±8.24, P=0.0012) versus controls. Lp(a) (OR=2.339,95%CI:1.519−3.603, P < 0.001) and CatLet© score (OR=1.092, 95%CI:1.027–1.161, P=0.005) independently predicted MACCE. The combined model Lp(a)⩾70.70 nmol/L + CatLet©⩾18.6) significantly outperformed individual markers (AUC 0.862 [95%CI:0.83−0.96] vs. 0.780/0.833; DeLong's test confirmed the superiority of the combined model over individual predictors (P = 0.0089, Z = 2.64 vs. Lp(a); P = 0.034, Z = 2.12 vs. CatLet© score), with 88% sensitivity and 83% specificity.

Conclusions: The Lp(a)-CatLet© synergy enhances MACCE risk stratification in ePCI-treated AMI, reflecting complementary *pathobiological* (Lp(a)-driven plaque vulnerability) and *anatomical* (CatLet©-quantified complexity) pathways. This dual-parameter approach could support post-PCI risk stratification and follow-up planning.

Keywords:

Lipoprotein(a); CatLet© coronary score; Acute myocardial infarction; Percutaneous coronary intervention; Major adverse cardiovascular and cerebrovascular events; Prognostic assessment

1.Introduction

Accurate risk stratification following emergency revascularization for AMI remains a critical challenge in contemporary cardiology practice. While current prognostic tools incorporating biochemical markers (e.g., cardiac troponins) and angiographic indices (e.g., SYNTAX score) provide modest predictive value(1, 2), their individual

limitations—including biological variability and anatomical oversimplification—underscore the need for more robust multimodal approaches(3, 4).

Emerging evidence implicates lipoprotein(a) (Lp(a)), a proatherogenic and prothrombotic particle, as a key mediator of post-PCI complications(5). Concurrently, the CatLet© scoring system (www.catletscore.com) has demonstrated superior discriminatory capacity for coronary lesion complexity compared to conventional scoring systems by incorporating dynamic weighting for bifurcation lesions, calcification, and functional significance(6).

We hypothesized that integrating Lp(a)'s pathobiological insights with CatLet©'s anatomical precision would optimize MACCE prediction. This investigation represents the first evaluation of this dual-parameter model in ePCI-AMI patients.

2. Materials and Methods

2.1 Study Design and Population

This retrospective cohort study included 307 consecutive acute myocardial infarction (AMI) patients undergoing successful emergency percutaneous coronary intervention (ePCI) at Bozhou People’s Hospital (China) between January 2020 and May 2022. AMI was diagnosed per the Fourth Universal Definition of Myocardial Infarction. Key exclusion criteria: prior MI, chronic heart failure (LVEF <40%), severe renal dysfunction (eGFR <30 mL/min/1.73m^2), or non-atherosclerotic coronary disease (e.g., vasculitis, cardiomyopathy). The study was conducted in accordance with the

Declaration of Helsinki. The protocol approval was obtained from the Medical Ethics Committee of Bozhou Hospital affiliated with Anhui Medical University(No. bz-2020-004). The identity of patients remained anonymous, and the requirement for informed consent was waived due to the retrospective anonymized data analysis.

2.2 Procedural and Pharmacological Protocols

All patients received guideline-directed therapy:

Pre-PCI: Loading doses of aspirin (300 mg) plus P2Y12 inhibitor (clopidogrel 300/600 mg or ticagrelor 180 mg).

Anticoagulation: Intravenous unfractionated heparin (100 IU/kg; ACT >300 sec), with glycoprotein IIb/IIIa inhibitors at operator discretion.

Post-PCI: Dual antiplatelet therapy (aspirin 100 mg/day + clopidogrel 75 mg/day or ticagrelor 90 mg twice daily) for ≥12 months, with high-intensity statins and beta-blockers as indicated.

High-risk support: Intra-aortic balloon pump (IABP) or IVUS-guided PCI for complex lesions (e.g., severe calcification, bifurcation).

2.3 Biomarker and Angiographic Analysis

Lp(a) quantification: Fasting serum Lp(a) was measured 24h post-admission via immunoturbidimetric assay (Roche; interassay CV <5%) on a Roche (cobas c 501 analyzer).(On the morning of the second day after admission, venous blood was drawn from the patient(7). Blood sample preparation involved drawing approximately 4 mL

of venous blood from the median cubital vein under sterile conditions, after the patient had fasted overnight. The sample was allowed to stand for 20 minutes before being centrifuged at 4000 rpm to separate the serum. The serum was then stored at -80°C for subsequent measurement of Lp(a) concentration.)

2.4 Angiographic scoring:

Gensini Score: Lesion severity was graded based on coronary stenosis degree, with segment-specific weighting factors applied. The total score was derived from the sum of individual lesion scores(8).

CatLet© Coronary Score: Angiographic complexity was assessed using the web-based CatLet© scoring calculator (available at http://www.catletscore.com). Only stenotic lesions were quantified(6, 9).

Follow-up

Follow-up was conducted via clinical visits and telephone interviews at 1, 6, and 12 months. Loss to follow-up was <5%. The censoring date was May 31, 2023**,** with yearly assessments continuing thereafter. A routine angiographic evaluation was advised at the 12-month mark following PCI. If patients experienced a return of angina symptoms, angiography was conducted earlier. For those who did not follow the recommended follow-up schedule, telephone interviews were conducted(10).

2.5 Endpoint Definitions(10, 11)

The main outcome of this research was a patient-focused composite measure of major adverse cardiac and cerebrovascular events (MACCE) at 1-year, encompassing all-cause mortality, myocardial infarction (MI), and revascularization of the target vessel

or lesion (TVR/TLR). When evaluating cumulative outcomes, each event was counted only once, prioritizing the first occurrence.

The secondary outcomes included a safety composite endpoint of all-cause death, MI, and stroke, alongside individual MACCE components and stent thrombosis (ST).

Death was categorized as any post-procedure fatality, classified into cardiac or non-cardiac based on the Academic Research Consortium (ARC) guidelines. A death was presumed to be of cardiac origin unless proven otherwise. Cardiac death encompassed fatalities due to cardiac issues (e.g., MI, low-output heart failure, lethal arrhythmias), those related to the procedure, or those of unknown origin.

Myocardial infarction (MI) was defined in accordance with the fourth universal MI definition(12), emphasizing elevated cardiac troponin (cTn) levels exceeding the 99th percentile upper reference limit (URL), symptoms of myocardial ischemia, ECG changes, and angiographic findings. Within 48 hours post-procedure, cTn levels exceeding 5 times the 99th percentile URL after PCI or 10 times after CABG indicated periprocedural MI in patients with normal baseline cTn levels. If baseline levels were elevated, stable, or decreasing, a cTn increase of over 20% also signified periprocedural PCI-related MI. Q-wave MI was identified by the presence of a new pathological Q-wave in at least two contiguous leads after initial treatment.

TVR was any surgical or percutaneous revascularization of any segment within the stented vessel (including target lesions and adjacent branches) within a year, such as the left main, left anterior descending, and left circumflex arteries. A pre-planned staged PCI was not deemed a TVR.

Definite stent thrombosis (ST) was defined per ARC criteria for PCI, while graft occlusion followed an ARC-like definition for CABG.

The definition of stroke is based on the currently internationally recognized criteria primarily derived from the expert consensus statement of the American Heart Association/American Stroke Association (AHA/ASA)(13).

Core Definition:

Stroke is defined as central nervous system (CNS) cell death accompanied by permanent injury, caused by ischemia or hemorrhage, based on neuropathological, neuroimaging, and/or clinical evidence.

Scope of Coverage:

This includes ischemic stroke (symptomatic CNS infarction), asymptomatic cerebral infarction (CNS infarction without known clinical symptoms), intracerebral hemorrhage, and subarachnoid hemorrhage.

2.6 Statistical Analysis

Continuous variables (mean±SD or median[IQR]) were compared via Student's *t*-test or Mann-Whitney U test; categorical variables (n[%]) via χ^2 or Fisher's exact test. Multivariable logistic regression (adjusted for age, sex, hypertension, diabetes, and LVEF) identified MACCE predictors. ROC analysis determined optimal Lp(a)/CatLet© cutoffs. Statistical significance was set at two-tailed $P<0.05$. Analyses used SPSS v26.0 (IBM).

3.Results

3.1 Baseline Characteristics

The MACCE group exhibited lower LVEF (51.6±11.7% vs. 54.9±12.0%, P=0.038), higher peak cTnI (4.86±2.13 vs. 4.35±1.56 ng/mL, P=0.023), and elevated Lp(a) (135.99±33.07 vs. 123.35±42.70 nmol/L, P=0.018) (Table 1).

3.2 Angiographic characteristics were comparable except for higher CatLet© (33.6±9.0 vs. 30.8±8.2, P=0.001) and Gensini scores (83.9±16.9 vs. 78.5±20.7, P=0.042) in the MACCE group (Table 2).

3.3 Multivariable logistic regression confirmed Lp(a) (OR = 2.339, P < 0.001) and CatLet© score (OR = 1.092, P = 0.005) as independent predictors of MACCE, along with LVEF, cTnI, and Gensini score (Table 3). Consistent results were observed in Cox proportional-hazards analysis (Table 4). Log-transformed Lp(a) as a continuous variable also showed a significant dose-response relationship with MACCE risk (Table 5).

3.4 Predictive Performance of Lp(a) and CatLet© Score for MACCE:in this cohort of AMI patients undergoing emergency PCI, both Lp(a) and the CatLet© coronary angiographic score demonstrated significant prognostic utility for MACCE: Individual Predictors: Lp(a):AUC: 0.780 (95% CI: 0.745–0.896),Optimal cutoff: 70.70 nmol/L (sensitivity 81.0%, specificity 76.5%, P<0.001),Clinical implication: Exceeds the EAS consensus high-risk threshold (125 nmol/L)(7), suggesting potential utility in risk stratification. CatLet© Score:AUC: 0.833 (95% CI: 0.820–0.927),Optimal cutoff: 18.6 points (sensitivity 83.0%, specificity 79.0%,

P<0.001),Anatomic correlation: Aligns with prior validation studies defining scores ⩾18 as indicative of complex coronary anatomy. Combined Model Superiority: The integration of Lp(a) and CatLet© score yielded enhanced predictive performance: AUC: 0.862 (95% CI: 0.830 – 0.960),Sensitivity/specificity: 88.0%/83.0% (P=0.009 vs. individual predictors, DeLong's test confirmed the superiority of the combined model over individual predictors (P = 0.0089, Z = 2.64 vs. Lp(a); P = 0.034, Z = 2.12 vs. CatLet© score).Net reclassification improvement (NRI): 0.35 (95% CI: 0.12 – 0.58; P=0.002),Statistical robustness: Bootstrap validation (1,000 iterations) confirmed model stability (mean AUC ± SD: 0.852 ± 0.021) (Table 6) (Figure 2). The combined model showed excellent calibration across risk deciles (Hosmer-Lemeshow P = 0.422; Table 7).

4. Discussion

This study demonstrates that the integration of lipoprotein(a) [Lp(a)] and the CatLet© coronary angiographic score significantly enhances the prediction of 1-year MACCE in AMI patients undergoing emergency PCI. The combined model outperformed either parameter alone, achieving an AUC of 0.862, with improved sensitivity and specificity. These findings underscore the complementary value of incorporating both a pathobiologic marker of plaque vulnerability and a comprehensive anatomic-functional scoring system into post-PCI risk stratification.

4.1. Synergistic Predictive Value of Lp(a) and CatLet© Score

The independent association of elevated Lp(a) (≥70.70 nmol/L) with MACCE aligns with its well-established proatherogenic, prothrombotic, and proinflammatory

properties (5, 14). Lp(a) promotes plaque instability through mechanisms including the inhibition of fibrinolysis, enhancement of foam cell formation, and induction of endothelial dysfunction. Concurrently, the CatLet© score provided a granular assessment of coronary lesion complexity, incorporating critical features such as bifurcation anatomy, calcification severity, and functional significance—elements not fully captured by traditional scores like SYNTAX or Gensini (6, 9, 15).

The superior predictive performance of the combined model suggests a synergistic interaction between systemic risk (reflected by Lp(a)) and localized anatomic burden (quantified by CatLet©). Patients with high Lp(a) may exhibit a heightened inflammatory and thrombotic milieu, which, when coupled with complex coronary anatomy, predisposes them to recurrent ischemic events. This dual-parameter approach thus bridges a critical gap between circulating risk biomarkers and coronary morphology.

4.2. Comparative Advantages of the CatLet© Score

Our results reinforce the clinical utility of the CatLet© score over conventional angiographic scoring systems. While the Gensini score focuses primarily on stenosis severity, and the SYNTAX score emphasizes lesion complexity, the CatLet© system incorporates dynamic weighting for calcification, angulation, and functional parameters such as TIMI flow—factors directly relevant to PCI difficulty and prognosis (6, 16). As shown in Table 8, the CatLet© score offers a more holistic evaluation, which may explain its stronger discriminatory capacity in our cohort.

4.3. Clinical and Mechanistic Implications

From a pathophysiological perspective, Lp(a) has been implicated in vascular calcification and fibrous cap thinning(17-19), processes that may exacerbate the anatomic complexity quantified by the CatLet© score. This interplay suggests that patients with elevated Lp(a) and high CatLet© scores may represent a high-risk phenotype warranting intensified management. This could include more aggressive lipid-lowering therapy (e.g., PCSK9 inhibitors), prolonged dual antiplatelet therapy, or closer follow-up—though such strategies require validation in prospective trials.

Moreover, the optimal Lp(a) cutoff of 70.70 nmol/L in our cohort is notably lower than the European Atherosclerosis Society consensus threshold of 125 nmol/L (7). This may reflect differences in study populations or the acute clinical context, highlighting the need for context-specific thresholds in AMI settings.

4.4. Limitations

Several limitations should be acknowledged. First, the single-center, retrospective design introduces potential selection bias. Second, although the CatLet© score has been validated in prior studies(6), its application requires specialized training and is not yet widely adopted. Third, Lp(a) assays are not fully standardized across platforms, which may affect the generalizability of the proposed cutoff(19). Finally, unmeasured confounders such as genetic predispositions or residual inflammatory risk may influence outcomes.

4.5. Future Directions

Prospective multicenter studies are needed to validate our findings and refine risk thresholds. Incorporating emerging biomarkers (e.g., high-sensitivity C-reactive

protein) or artificial intelligence–based plaque characterization could further enhance predictive models(20). Additionally, randomized trials are warranted to assess whether risk-guided therapeutic interventions improve outcomes in this high-risk subgroup.

4.6. Conclusion

In conclusion, the combination of Lp(a) and the CatLet© score provides a robust, clinically feasible tool for stratifying MACCE risk in AMI patients after emergency PCI. This integrated model captures both systemic lipid-related risk and coronary anatomic complexity, offering a more comprehensive prognostic assessment than either measure alone. With further validation, this approach could inform personalized secondary prevention strategies and follow-up protocols.

Funding

This study was supported by the National Natural Science Foundation of China (Grant No. 82170333), Bozhou City Key R&D Program of Anhui Province of China (No. bzzc2024004 & bzzc2024001), Medical Science and Technology Research Project of Henan Province (Grant No. SBGJ202102153), Henan Provincial Science and Technology Research Projects (Grant No. 212102310799 and 222102310577).

Conflict of Interest statement

All authors have no conflicts of interest.

Reference

1. Ananthan K, Lyon AR. The Role of Biomarkers in Cardio-Oncology. J Cardiovasc Transl Res. 2020;13(3):431-50.

2. Birnbaum Y, Herz I, Sclarovsky S, Zlotikamien B, Chetrit A, Olmer L, et al. Prognostic

significance of precordial ST segment depression on admission electrocardiogram in patients with inferior wall myocardial infarction. J Am Coll Cardiol. 1996;28(2):313-8.

3. Gholipour A, Shakerian F, Zahedmehr A, Irani S, Malakootian M, Mowla SJ. Bioinformatics Analysis to Find Novel Biomarkers for Coronary Heart Disease. Iran J Public Health. 2022;51(5):1152-60.

4. V R, Rao S, Nayak K, M M, Samanth J, R P. Assessing angiographic results of saline autotransfusion and thrombus aspiration in STEMI patients. Future Cardiol. 2025;21(4):237-43.

5. Minana G, Cordero A, Facila L, Company M, Fernandez-Cisnal A, Valero E, et al. Lipoprotein(a) and Long-Term Recurrent Infarction After an Acute Myocardial Infarction. Am J Cardiol. 2024;211:9-16.

6. He YM, Masuda S, Gao C. Revisit of the CatLet (Hexu) angiographic scoring system: a narrative review. J Thorac Dis. 2023;15(5):2848-58.

7. Mach F, Baigent C, Catapano AL, Koskinas KC, Casula M, Badimon L, et al. 2019 ESC/EAS Guidelines for the management of dyslipidaemias: lipid modification to reduce cardiovascular risk. European heart journal. 2020;41(1):111-88.

8. Rampidis GP, Benetos G, Benz DC, Giannopoulos AA, Buechel RR. A guide for Gensini Score calculation. Atherosclerosis. 2019;287:181-3.

9. Xu M, Wang S, Zhang Y, Zhang J, Ma J, Shen J, et al. Residual coronary artery tree description and lesion EvaluaTion (CatLet) score, clinical variables, and their associations with outcome predictions in patients with acute myocardial infarction. Chin Med J (Engl). 2023;136(20):2459-67.

10. Tu S, Zhang L, Tian Q, Hu F, Wang Y, Chen L. Five-year outcomes of double kissing mini-culotte stenting vs. mini-culotte stenting using drug-eluting stents for the treatment of true coronary bifurcation lesions. Frontiers in cardiovascular medicine. 2024;11:1336750.

11. Lee SH, Hong D, Shin D, Kim HK, Park KH, Choo EH, et al. QFR Assessment and Prognosis After Nonculprit PCI in Patients With Acute Myocardial Infarction. JACC Cardiovascular interventions. 2023;16(19):2365-79.

12. Thygesen K, Alpert JS, Jaffe AS, Chaitman BR, Bax JJ, Morrow DA, et al. Fourth Universal Definition of Myocardial Infarction (2018). Circulation. 2018;138(20):e618-e51.

13. Powers WJ, Rabinstein AA, Ackerson T, Adeoye OM, Bambakidis NC, Becker K, et al. Guidelines for the Early Management of Patients With Acute Ischemic Stroke: 2019 Update to the 2018 Guidelines for the Early Management of Acute Ischemic Stroke: A Guideline for Healthcare Professionals From the American Heart Association/American Stroke Association. Stroke. 2019;50(12):e344-e418.

14. Ting Q, Tian-Yi M, Kang H, Shi-Juan L, Jiang-Hua Z, Jian-Jun LJCAR. Lipoprotein (a)-Related Inflammatory Imbalance: A Novel Horizon for the Development of Atherosclerosis. 2024;26(8).

15. Ming-Xing X, Ruo-Ling T, Terrence D R, Paul S, Thomas B, Roberto DB, et al. The CatLet score: a new coronary angiographic scoring tool accommodating the variable coronary anatomy for the first time. 2020;11(12).

16. Lan-Lin W, Jian-Ping X, Yang H, Heng W, Guo-Zhong Z, Kai W, et al. Coronary Artery Tree Description and Lesion Evaluation (CatLet) score for functional evaluation of

coronary stenosis: a comparison study with pressure wire fractional flow reserve. 2024;14(4).

17. Laura A-H, Julián A-J, Jacob Fog BJFCM. Mechanisms of fibrous cap formation in atherosclerosis. 2023;10(0).

18. Marlys L K, Michael B BJA. Oxidized phospholipid modification of lipoprotein(a): Epidemiology, biochemistry and pathophysiology. 2022;349(0).

19. Jia P, Ming-Ming L, Hui-Hui L, Rui-Xia X, Cheng-Gang Z, Yuan-Lin G, et al. Lipoprotein (a)-mediated vascular calcification: population-based and in vitro studies. 2021;127(0).

20. Jacek KJJCI. Artificial Intelligence-Based Quantitative Coronary Plaque Analysis. 2023;17(3).

Figure 1 : Research Flowchart for the Study Click here to access/download;Figure;Figure 1.tif

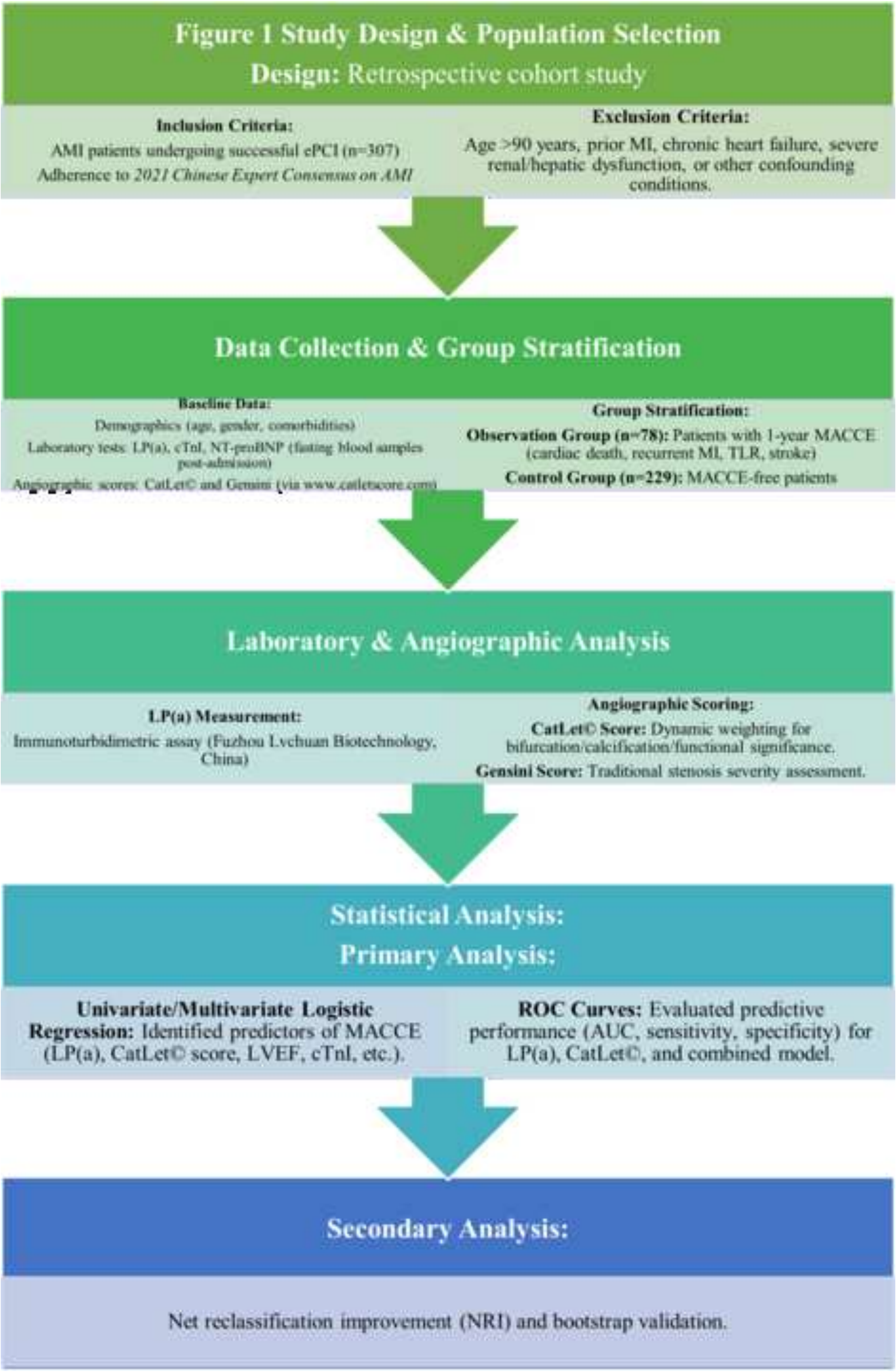

Figure 2 : ROC Analysis

Click here to access/download;Figure;Figure 2.tiff

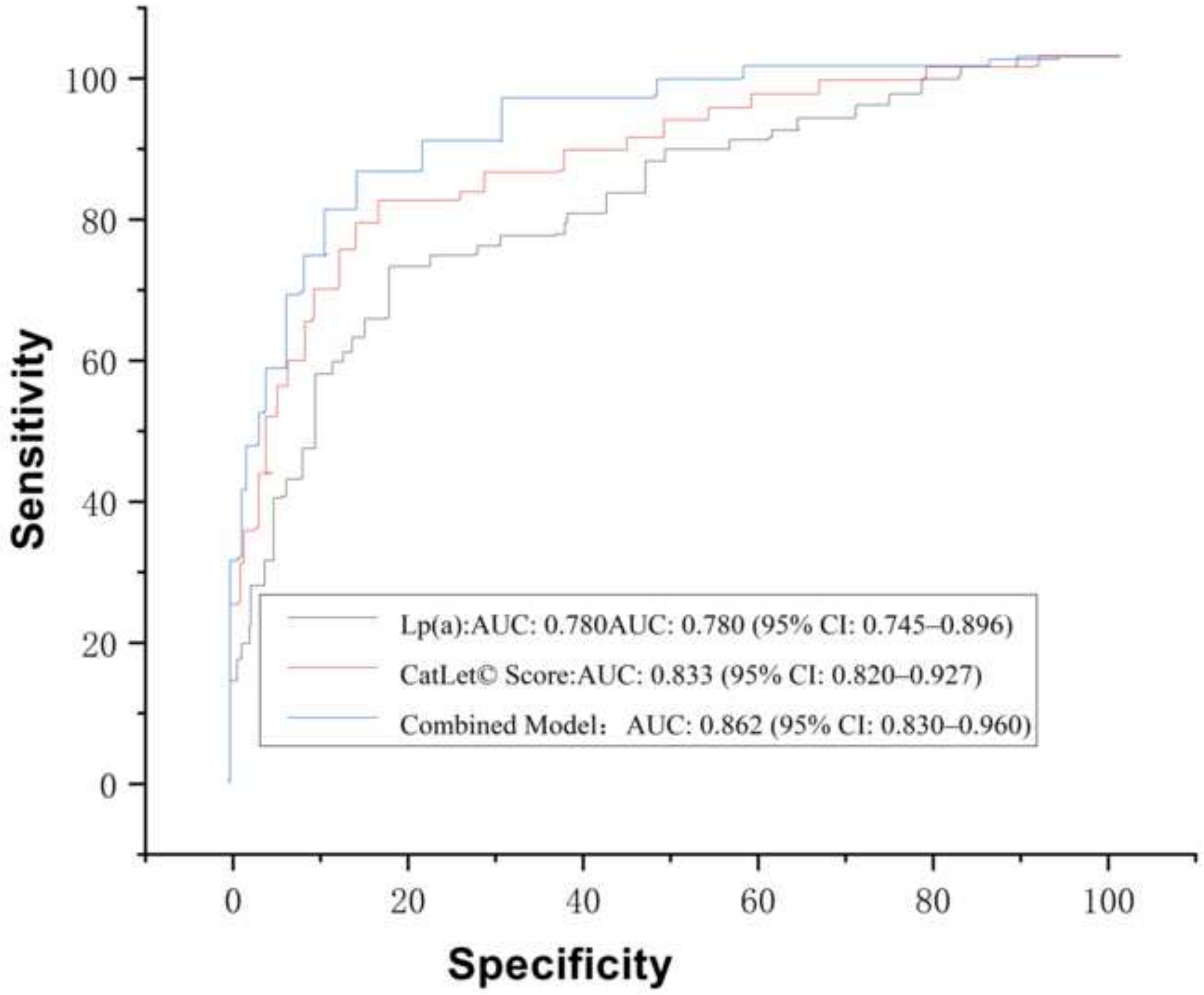

Figure legends Click here to access/download;Figure;Figure Legends.docx

Figure legends

Figure 1: Research Flowchart for the Study: "Clinical Value of Lipoprotein(a) Combined with CatLet© Coronary Score in Predicting Adverse Events after Emergency PCI for AMI Patients"

Figure 2: Figure Legend: ROC Analysis of Lp(a) and CatLet Score for 1-Year MACCE Prediction Post-AMI:

The predictive performance of Lp(a) and the CatLet© score is demonstrated through ROC curve analysis in a cohort of AMI patients (n=307).Lp(a): AUC of 0.780 (95% CI: 0.745–0.896) with an optimal cutoff of 70.70 nmol/L, sensitivity 81.0%, specificity 76.5% (P<0.001). This cutoff exceeds the EAS consensus high-risk threshold (125 nmol/L), indicating its utility in risk stratification.CatLet© Score: AUC of 0.833 (95% CI: 0.820–0.927) with an optimal cutoff of 18.6 points, sensitivity 83.0%, specificity 79.0% (P<0.001). Scores ≥18 correlate with complex coronary anatomy based on prior validation studies.Combined Model: The integration of Lp(a) and CatLet© score yielded an AUC of 0.862 (95% CI: 0.830–0.960), sensitivity 88.0%, specificity 83.0% (P=0.009 vs. individual predictors). Net reclassification improvement (NRI) was 0.35 (95% CI: 0.12–0.58; P=0.002), and bootstrap validation confirmed model stability with mean AUC ± SD of 0.852 ± 0.021.

table 1

Click here to access/download
Supporting Information
Table 1.docx

table 2

Click here to access/download
Supporting Information
Table 2.DOCX

table 3

Click here to access/download
Supporting Information
Table 3.docx

table 4

Click here to access/download
Supporting Information
Table 4.docx

table 5

Click here to access/download
Supporting Information
Table 5.docx

table 6

Click here to access/download
Supporting Information
Table 6.docx

table 7

Click here to access/download
Supporting Information
Table 7.docx

table 8

Click here to access/download
Supporting Information
Table 8.DOCX
